# Extending the ‘one-point method’ for estimations of leaf photosynthetic capacity to a broader temperature range

**DOI:** 10.1093/jxb/erac466

**Published:** 2022-11-26

**Authors:** Tony César de Sousa Oliveira, Maquelle Neves Garcia, Elmar Veenendaal, Tomas Ferreira Domingues

**Affiliations:** FFCLRP, Department of Biology, University of São Paulo (USP), Ribeirão Preto, Brazil; Plant Ecology and Nature Conservation Group, Wageningen University and Research (WUR), Wageningen, The Netherlands; National Institute for Amazonian Research (INPA), Manaus, Brazil; Plant Ecology and Nature Conservation Group, Wageningen University and Research (WUR), Wageningen, The Netherlands; FFCLRP, Department of Biology, University of São Paulo (USP), Ribeirão Preto, Brazil; University of Cambridge, UK

**Keywords:** Global vegetation models, global warming, photosynthesis, temperature response, *V*_cmax_

## Abstract

We propose a temperature-dependent scaling factor for the ‘one-point method’ to mitigate the overestimation of the maximum carboxylation capacity of photosynthesis at high temperatures.

**The ‘one-point method’ (OPM) is a quick approach to estimate the maximum apparent carboxylation rate of Rubisco (*Vʹ***_**cmax**_ **) based on a single measurement of leaf carbon assimilation rate taken under saturating light and ambient CO**_**2**_ **, lately equal to 400 ppm (** De Kauwe *et al.*, 2016 **). However, the OPM overestimates *Vʹ***_**cmax**_ **at high temperatures (** Burnett *et al.*, 2019 **). This overestimation results from the reliance of the method on a linear relationship between *Vʹ***_**cmax**_ **and leaf respiration rates under light (*R***_**day**_ **), as a fixed *R***_**day**_ **:*V***_**cmax**_ **ratio equal to 1.5%, while these parameters have different temperature dependencies. Here, we highlight the importance of the adoption of a temperature-dependent scaling factor for the *R***_**day**_ **:*V***_**cmax**_ **ratio as a way to prevent the overestimation of the photosynthetic capacity at temperatures >35 °C.**

The maximum carboxylation rate of Rubisco (*V*_cmax_) is a key photosynthetic enzyme characteristic that reflects plant individual fitness. It is also a prescribed variable of global vegetation models (GVMs), which calculate primary productivity of terrestrial vegetation. This parameter is usually estimated from CO_2_ response curves (*A*–*C*_i_ curves, where *C*_i_ is intercellular [CO_2_]) (e.g. Farquhar *et al.*, 1980—the FvCB model). Recently, the OPM was proposed as a fast alternative to standard full biochemical curve-fitting methods (De Kauwe *et al.*, 2016). Against the gold-standard *A*–*C*_i_ curves, the OPM has proven to be very useful as it allows for the characterization of a large number of species and individual leaves in a short period of time (5–10 min per individual), enabling a better parameterization of highly diverse tropical vegetation communities within GVMs. Another alternative available is the rapid *A*–*C*_i_ technique (RACiR) (Stinziano *et al.*, 2017), which upon any change in conditions, such as leaf temperature, requires some data processing after the measurements are taken. Apart from being fast, another advantage of the OPM is the minimization of negative effects of performing multiple full *A*–*C*_i_ curves on the same leaf in order to obtain the temperature dependency of photosynthetic parameters. Therefore, providing measurements are taken with the proper care and allowing enough time for stabilization of gas exchange fluxes, the OPM has the potential to significantly improve our ability to describe the carbon uptake strategies of highly diverse plant communities.

According to De Kauwe *et al.* (2016), *Vʹ*_cmax_ can be calculated as:


$$\begin{array}{l} V{\;\overset{´}{\;}\;}_{cmax}=\left(A_{sat}+R_{day}\right)\times \frac{\left(C_{i}+K_{m}\right)}{\left(C_{i}-{\;\Gamma \;}^{*}\;\;\;\right)} \end{array}$$ (1)

Where *C*_i_ is the intercellular CO_2_ concentration, Γ* is the CO_2_ compensation point in the absence of mitochondrial respiration, *K*_m_ is the Michaelis–Menten constant of Rubisco, and *R*_day_ is the leaf mitochondrial respiration in the light. When *R*_day_ is not determined experimentally, the authors suggest the use of an estimated *R*_day_ ratio equal to 1.5% of *Vʹ*_cmax_ (0.015 in Equation 2).


$$\begin{array}{l} V{\;\overset{´}{\;}\;}_{cmax}=\frac{A_{sat}}{\left(\;\;\;\frac{C_{i}\;\;\;-\;\;\;{\;\Gamma \;}^{*}}{C_{i}\;\;\;+\;\;\;K_{m}}-0.015\right)} \end{array}$$ (2)

However, the OPM provides an overestimation of *Vʹ*_cmax_, especially at high temperatures above 30 °C (De Kauwe *et al.* 2016; Slot and Winter, 2017; Burnett *et al.* 2019). As the temperature dependencies of *R*_day_ and *V*_cmax_ differ considerably from each other (Wang *et al.* 2020), assuming a linear relationship between these parameters as a constant ratio results in the overestimation of *Vʹ*_cmax_, as *R*_day_ peaks at a much higher leaf temperature. A better accuracy of the OPM is needed to improve its performance at higher leaf temperatures. In this sense, we propose here the adoption of a temperature-dependent scaling factor for the *R*_day_:*Vʹ*_cmax_ ratio in order to improve predictions of *Vʹ*_cmax_.

The temperature dependencies of both *R*_day_ and *V*_cmax_ may be defined by standard Arrhenius functions, following Kumarathunge *et al.* (2019) as:


$$\begin{array}{l} \frac{{R_{day}}^{T}}{{R_{day}}^{R}}=e\;\;\;\frac{Ea_{R}\;\;\;\times \;\;\;\left(T_{k}-298.15\right)}{298.15\;\;\;\times \;\;\;R\;\;\;\times \;\;\;T_{k}} \end{array}$$ (3)

and


$$\begin{array}{l} \frac{{V_{cmax}}^{T}}{{V_{cmax}}^{R}}=e\;\;\;\frac{Ea_{V}\times \left(T_{k}-298.15\right)}{298.15\times R\times T_{k}}\times \;\;\;\left[\frac{1+\;e{\;\;\;}^{\left(\frac{298.15\times \Delta S_{V}\;-\;Hd_{V}}{298.15\;\;\;\times \;\;\;R}\right)}}{1+e^{\left(\frac{(T_{k\;}\times \;\Delta S_{V}\;-\;Hd_{V}}{T_{k}\times \;R}\right)}}\right] \end{array}$$ (4)

where *R*_day_^T^ and *V*_cmax_^T^ are respiration and carboxylation rates at a given leaf temperature (*T*_k_ in Kelvin), *R*_day_^R^ and *V*_cmax_^R^ are the rates at a reference leaf temperature of 25 °C, R is the universal gas constant (8.314 J mol^−1^ K^−1^), Ea_R_ (kJ mol^−1^) is the activation energy for respiration, and Ea_V_ (kJ mol^−1^), ΔS_V_ (J mol^−1^ K^−1^), and Hd_V_ (kJ mol^−1^) are respectively the activation energy, entropy, and deactivation energy of *V*_cmax_. The adopted values for model constants are available in Supplementary Table S1.

Our new suggested model modifies Equation 2 for:


$$\begin{array}{l} V{\;\overset{´}{\;}\;}_{cmax-\rho }=\frac{A_{sat}}{\left(\;\;\;\frac{C_{i}-{\;\Gamma \;}^{*}}{C_{i}+K_{m}}-0.015\times \rho \right)} \end{array}$$ (5)

where ρ is the temperature-dependent scaling on the *R*_day_ ratio (1.5% of *Vʹ*_cmax_), which is calculated by dividing Equation 3 by Equation 4.

The robustness of this approach was tested using 278 unpublished *A*–C_i_ curves (12 CO_2_ concentration steps) under saturating light (2000 μmol m^–2^ s^–1^), collected from 31 tropical species naturally occurring in savannas and Amazon rainforest in Brazil (Supplementary Table S2) using two portable gas exchange systems (LI-6800, Li-Cor Inc., Lincoln, NE, USA). Leaf temperatures were kept constant during each curve, but ranged from 25 °C to 45 °C among curves, and the leaf chamber relative humidity was not controlled. Over the whole dataset, the leaf-to-air vapor pressure deficit ranged from 1.08 kPa to 7.71 kPa (3.84 ± 1.65), while stomatal conductance ranged from 0.05 mol m^−2^ s^−1^ to 0.34 mol m^−2^ s^−1^ (0.134 ± 0.06). To estimate *Vʹ*_cmax_ and *Vʹ*_cmax–ρ_ values, we selected a single point measurement from each *A*–*C*_i_ curve, corresponding to ambient CO_2_ concentrations (*C*_a_) between 390 μmol mol^–1^ and 410 μmol mol^–1^. The *K*c, *K*o, and Γ* values used in Equations 2 and 3 were calculated according to the temperature dependencies listed in Bernacchi *et al.* (2002) and De Kauwe *et al.* (2016). Linear regressions were used to compare both *Vʹ*_cmax_ and *Vʹ*_cmax–ρ_ with *V*_cmax_ from full *A*–*C*_i_ curves (Duursma, 2015). The slopes between the two linear regressions were compared using ANOVA. Moreover, the distribution of the residuals of the regression models as a function of leaf temperature were used for method accuracy comparisons. Additionally, a multivariate sensitivity analysis was performed to investigate the role of temperature over the range values of the Arrhenius constants regarding the model outputs. All analyses we performed on R environment (R Core Team, 2020).

The temperature-dependent scaling factor provided a substantially better correspondence between *V*_cmax_ values from FvCB fits against the *Vʹ*_cmax–ρ_ values, when compared with *Vʹ*_cmax_ values from the original OPM (Fig. 1) (slope=1.10; *r*^2^=0.93). The ANOVA test revealed that the outputs from the two models were statistically different (*P*<0.005). The evaluation of the distribution of the residuals from the two regression models highlights how the overestimation of *Vʹ*_cmax_ by the original version of the OPM increases with leaf temperature (Fig. 2A), where overestimations of ~25% were observed above 35 °C. In contrast, no trend was observed for the residuals from the new model in relation to leaf temperature (Fig. 2B). The adoption of the Arrhenius temperature dependency for *R*_day_ (Equation 3) was preferred over a Q_10_ temperature coefficient (Atkin *et al.*, 2015), the latter resulting in an overestimation of *Vʹ*_cmax–ρ_ values of ~25% (slope=1.29, *r*^2^=0.85) (Supplementary Fig. S1), with a non-significant difference between its slope against the model using the the original OPM approach (*P*=0.69). The sensitivity analysis revealed that the temperature-dependent scaling factor is strongly influenced by both Ea_R_ and Ea_V_ above 25°C (Supplementary Fig. S2). As temperature increases, their relative importance decreases, while ΔS_V_ and the interaction among parameters become the main factors determining *R*_day_:*V*_cmax_.

**Fig. 1.**
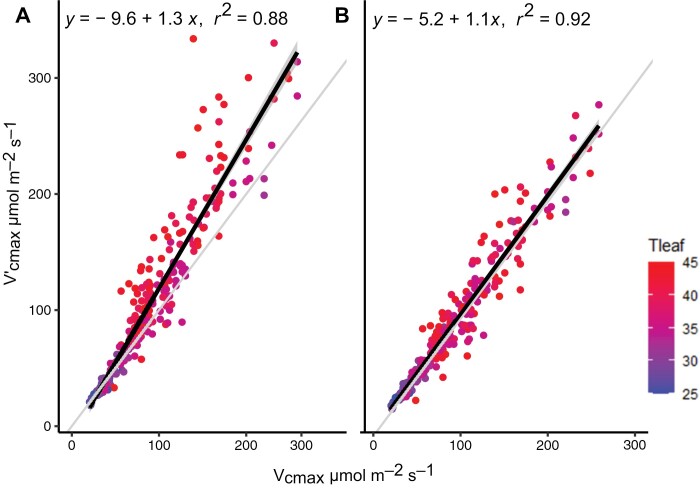
Comparison of linear regression models between *V*_cmax_ estimated from full *A*–*C*_i_ curves against apparent photosynthetic capacity estimated by the ‘one-point method’ (*Vʹ*_cmax_; Equation 2) (A), and the modified version including the temperature dependency (*Vʹ*_cmax–ρ_; Equation 5) (B). The light gray line is the 1:1 relationship.

**Fig. 2.**
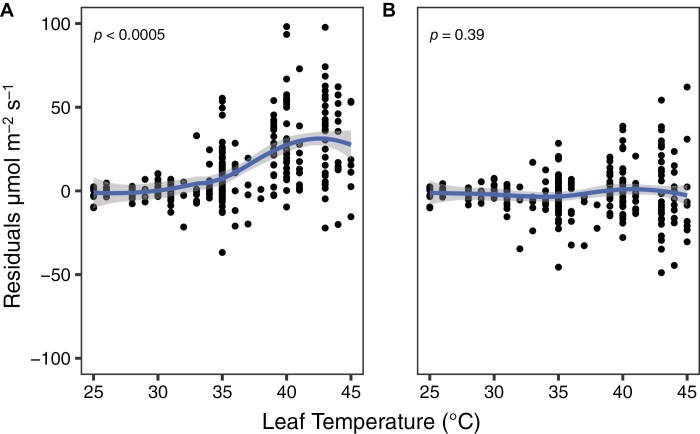
Residuals of maximum carboxylation rate, *V*_cmax_, estimated from *A*–*C*_i_ curves estimated from apparent maximum carboxylation capacity, *Vʹ*_cmax_ (Equation 2) using the estimated *R*_day_:*V*_cmax_ ratio (A), and using the temperature-dependent scaling factor (B) as a function of leaf temperature.

The adoption of a temperature-dependent scaling is indeed an important adjustment of the OPM, producing accurate estimations of *Vʹ*_cmax_ at high temperatures where there is proportionality of *Vʹ*_cmax_ and *R*_day_ responses to temperature (Wang *et al.*, 2020), as demonstrated empirically in Supplementary Fig. S3. Therefore, the temperature-dependent scaling factor proposed here should allow for better determinations of the temperature response of *Vʹ*_cmax_. However, providing reliable data for derivations of the Arrhenius constants is needed, as these constants vary among functional types of plants and species (Kumarathunge *et al.*, 2019). Apart from the difficult task of adopting appropriate values for the Arrhenius constants for *R*_day_ and *V*_cmax_, other aspects might be important as well. The application of biochemical models of photosynthesis often disregards the influence of *g*_m_ over estimations of *Vʹ*_cmax_, assuming it to be large enough to cause *C*_i_ to be equal to chloroplastic CO_2_ concentration (*C*_c_). However, *g*_m_ varies with leaf temperature as a result of both enzymatic dynamics and CO_2_ diffusion, resulting in an overestimation of *V*_cmax_ when assuming an infinite *g*_m_ (von Caemmerer and Evans, 2015), therefore directly impacting the *R*_day_:*V*_cmax_ ratio. Similarly, the temperature dependency of the Γ* is assumed to be invariant among plant species, and a single function is often used to scale this parameter to a specific leaf temperature (Bernacchi *et al.*, 2002). However, depending on the Γ* assumption, the relationship between temperature and the *R*_day_:*V*_cmax_ ratio may change (De Kauwe *et al.*, 2016); this is because the stoichiometry of CO_2_ release by Rubisco oxygenation depends on how Γ* responds to temperature (Bernacchi *et al.*, 2002). Therefore, a better understanding of possible variations of the temperature dependencies of both *g*_m_ and Γ* is still needed in order to improve the understanding of temperature influence on the estimations of *Vʹ*_cmax_, irrespective of the estimation method used.

In conclusion, the inclusion of a temperature-dependent scaling factor in the ‘one-point method’ extends its applicability to leaf temperatures >35 °C. Therefore, this approach should contribute to the characterization of vegetation communities and provide data for the estimation of Arrhenius parameters without stressing leaves by performing repeated CO_2_ response curves. Efforts should be directed to the understanding of the variation of temperature dependencies of the photosynthetic parameters, specifically the entropy and the activation and deactivation energies of *V*_cmax_, *R*_day_, and also the maximum electron transport rate (*J*_max_).

## Supplementary data

The following supplementary data are available at *JXB* online.

Table S1. Dataset of primary parameters and their temperature dependency used to estimate *R*_day_ and *Vʹ*_cmax_ temperature response in Equations 3 and 4.

Table S2. Species studied with the biome, their family, number of individuals (N individuals) and curves (N curves), and temperature range curves.

Fig. S1. Comparison of linear regression models between *V*_cmax_ estimated from full *A*–*C*_i_ curves against apparent photosynthetic capacity estimated by the ‘one-point method’.

Fig. S2. Normalized partitioning of the variation of the influence of individual coefficients over model output at a broad leaf temperature range (sensitivity analysis).

Fig. S3. Estimated *R*_day_ (*R*_day_:*V*_cmax_ ratio) as a function of leaf temperature using the De Kauwe *et al.* (2016) model.

erac466_suppl_Supplementary_Tables_S1-S2_Figures_S1-S3

## Data Availability

The data supporting the findings of this study are available from the corresponding author, Tony de Oliveira, upon request.

## References

[CIT0001] Atkin OK , Bloomfield KJ, Reich PB, et al. 2015. Global variability in leaf respiration in relation to climate, plant functional types and leaf traits. New Phytologist 206, 614–636. 25581061 10.1111/nph.13253

[CIT0002] Bernacchi, CJ, Portis AR, Nakano H, Caemmerer S, Long SP. 2002. Temperature response of mesophyll conductance. implications for the determination of rubisco enzyme kinetics and for limitations to photosynthesis in vivo. Plant Physiology 130, 1992–1998. 12481082 10.1104/pp.008250 PMC166710

[CIT0003] Burnett AC , Davidson KJ, Serbin SP, Rogers A. 2019. The ‘one-point method’ for estimating maximum carboxylation capacity of photosynthesis: a cautionary tale. Plant, Cell & Environment 42, 2472–2481. 10.1111/pce.13574 31049970

[CIT0004] De Kauwe MG , Lin YS, Wright IJ, et al. 2016. A test of the ‘one-point method’ for estimating maximum carboxylation capacity from field-measured, light-saturated photosynthesis. New Phytologist 210, 1130–1144. 26719951 10.1111/nph.13815

[CIT0005] Duursma RA. 2015. Plantecophys—an R package for analysing and modelling leaf gas exchange data. PLoS One 10, e01433461–e01433413. 10.1371/journal.pone.0143346 PMC4651500 26581080

[CIT0006] Farquhar GD , Von Caemmerer S, Berry JA. 1980. A biochemical-model of photosynthetic CO_2_ assimilation in leaves of C_3_ species. Planta 149, 78–90. 24306196 10.1007/BF00386231

[CIT0007] Kumarathunge DP , Medlyn BE, Drake JE, et al. 2019. Acclimation and adaptation components of the temperature dependence of plant photosynthesis at the global scale. New Phytologist 22, 768–784. 10.1111/nph.15668 30597597

[CIT0008] R Core Team. 2020. R: a language and environment for statistical computing. Vienna, Austria: R Foundation for Statistical Computing. https://www.R-project.org/.

[CIT0009] Slot M , Winter K. 2017. *In situ* temperature relationships of biochemical and stomatal controls of photosynthesis in four lowland tropical tree species. Plant, Cell & Environment 40, 3055–3068. 10.1111/pce.13071 28926102

[CIT0010] Stinziano JR , Morgan PB, Lynch DJ, et al. 2017. The rapid A–*C*i response: photosynthesis in the phenomic era. Plant, Cell & Environment 40, 1256–1262. 10.1111/pce.12911 28247953

[CIT0011] Wang H , Atkin OK, Keenan TF, Smith NG, Wright IJ, Bloomfield KJ, Kattge J, Reich PB, Prentice IC. 2020. Acclimation of leaf respiration consistent with optimal photosynthetic capacity. Global Change Biology 26, 2573–2583. 10.1111/gcb.14980 32091184

[CIT0012] von Caemmerer S , Evans JR. 2015. Temperature responses of mesophyll conductance. Plant, Cell & Environment 38, 629–637. 10.1111/pce.12449 25224884

