# Supplementary material for: Extending the ‘one-point method’ for estimations of leaf photosynthetic capacity to a broader temperature range
Source: J Exp Bot. 2022 Nov 26;74(3):684–7. doi: 10.1093/jxb/erac466 (PMC9899412; doi:10.1093/jxb/erac466)
Supplement: erac466_suppl_Supplementary_Tables_S1-S2_Figures_S1-S3 [file erac466_suppl_supplementary_tables_s1-s2_figures_s1-s3.pdf]

Supplementary Material

Extending the 'One-point method' for estimations of leaf photosynthetic capacity to a broader temperature range

Tony César de Sousa Oliveira[1,2*], Maquelle Neves Garcia[3], Elmar Veenendaal[2], Tomas Ferreira Domingues[1]

[1] FFCLRP, Department of Biology, University of São Paulo (USP), Ribeirão Preto, Brazil

[2] Plant Ecology and Nature Conservation, Wageningen University and Research (WUR), Wageningen, The Netherlands

[3] National Institute for Amazonian Research (INPA), Manaus, Brazil. E-mail

*Correspondence: tonycsoliveira@usp.br

Supplementary Table S1: Data set of primary parameters and their temperature dependency used to estimate V'_{cmax} and R_{day} temperature response in of Eqns 3 and 4. Where R is the universal gas constant; Ea_V, ΔS_V, and H_{dV} are respectively the activation energy, as entropy and deactivation energy of V'_{cmax}, and Ea_R is the activation energy of R_{day}

Parameter	Units	Values used here
R	$J\ mol^{-1}\ K^{-1}$	8.314 [1]
Ea_R	$kJ\ mol^{-1}$	20700[2]
Ea_V	$kJ\ mol^{-1}$	58550[1]
ΔSV	$Jmol^{-1}K^{-1}$	629.26[1]
Hd_V	$kJ\ mol^{-1}$	20000[1]

[1]Kumarathunge, Medlyn and Duursma (2018)

[2] Kumarathunge et al (2018)

Supplementary Table S2: Species studied accordingly with the biome, their family, number of individuals (N curves) and curves (N curves), and temperature range curves.

Biome	Species	Family	N individuals	N curves	Leaf Temperature range
Savanna					
	Xylopia aromatica (Lam.) Mart. LC	Annonaceae	3	9	35°-43°C
	Vochysia tucanorum Mart	Vochysiaceae	3	9	35°-43°C
	Stryphnodendron adstringens (Mart.)	Fabaceae	3	9	35°-43°C
	Qualea grandiflora Mart.	Vochysiaceae	6	18	35°-43°C
	Qualea parviflora Mart.	Vochysiaceae	3	9	35°-43°C
	Ormosia arborea (Vell.) Harms Coronheira	Fabaceae	3	9	35°-43°C
	Hymenaea stigonocarpa Mart. ex Hayne	Fabaceae	6	18	35°-43°C
	Annona coriacea Mart.	Annonaceae	6	18	35°-43°C
	Vatairea macrocarpa (Benth.) Ducke	Fabaceae	3	9	35°-43°C
	Stryphnodendron coreacium (Mart.)	Fabaceae	3	9	35°-43°C
	Psidium myrsinoides. O.Berg.	Myrtaceae	3	9	35°-43°C
	Oxandra sessiliflora R.E.Fr. |	Annonaceae	3	9	35°-43°C
	Himatanthus obovatus (Muell.Arg.) Woodson	Apocynaceae	3	9	35°-43°C

	Caryocar coriaceum Wittm. LC	Caryocaraceae	3	9	35°-43°C
Amazonia					
	Pterandra arborea Ducke	Malpighiaceae	1	5	25°-45°C
	Licania coriacea Benth	Chrysobalanaceae	1	5	25°-45°C
	Vantanea parviflora Lam	Humiriaceae	1	8	25°-45°C
	Pouteria erythrochrysa T.D.Penn	Sapotaceae	1	5	25°-45°C
	Diploon cuspidatum (Hoehne) Cronquist	Sapotaceae	1	5	25°-45°C
	Matayba purgans Radlk.	Sapindaceae	1	5	25°-45°C
	Pourouma tomentosa C.Mart. ex Miq	Urticaceae	1	5	30°-45°C
	Pouteria minima T.D.Penn	Sapotaceae	1	6	25°-45°C
	Ocotea cernua (Nees) Mez	Lauraceae	1	5	25°-45°C
	Protium ferrugineum (Engl.) Engl	Burseraceae	1	5	25°-45°C
	Eschweilera coriacea (DC.) S. A	Lecythidaceae	1	4	30°-45°C
	Pouteria caimito (Ruiz et Pavon) Radlk.	Sapotaceae	1	5	25°-45°C
	Sloanea fragrans Rusby	Elaeocarpaceae	1	6	25°-45°C
	Mabea angularis Hollander	Euphorbiaceae	1	5	25°-45°C
	Eschweilera grandiflora (Aubl.) Sandwith	Lecythidaceae	1	5	25°-45°C

Pouteria platyphylla (A.C.Sm.) Baehni	Sapotaceae	1	5	25°-45°C
Duguetia stelechantha (Diels) REFr.	Annonaceae	1	5	25°-45°C
Protium hebetatum Daly	Burseraceae	1	5	25°-45°C
Cordiera myrciifolia (K.Schum.) C.H.Perss. & Delprete.	Rubiaceae	1	5	25°-45°C

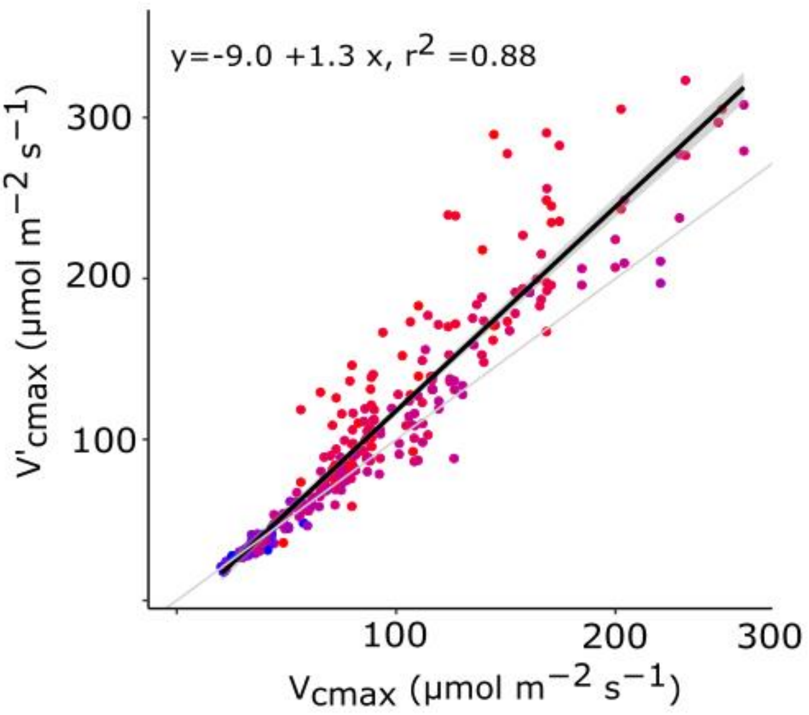

Supplementary Figure S1: Comparison of linear regression models between V_{cmax} estimated from full A-C_i curves against apparent photosynthetic capacity estimated by the "One-point method" (V'_{cmax} - Eqn 2), using the temperature-dependent Q10 using the equation: ${R_{day}}^{T}/{R_{day}}^{R} = R_{25} * {Q_{10}}^{(\frac{T-25}{10})}$ as the numerator in Eqn 5, where R_{25} represents the respiratory rates at 25 °C, T is the leaf temperature and Q_{10} represents the factor by which the respiratory rate changes with a 10 °C increase in temperature (Atkin et al 2015). The light-gray line is the 1:1 relationship.

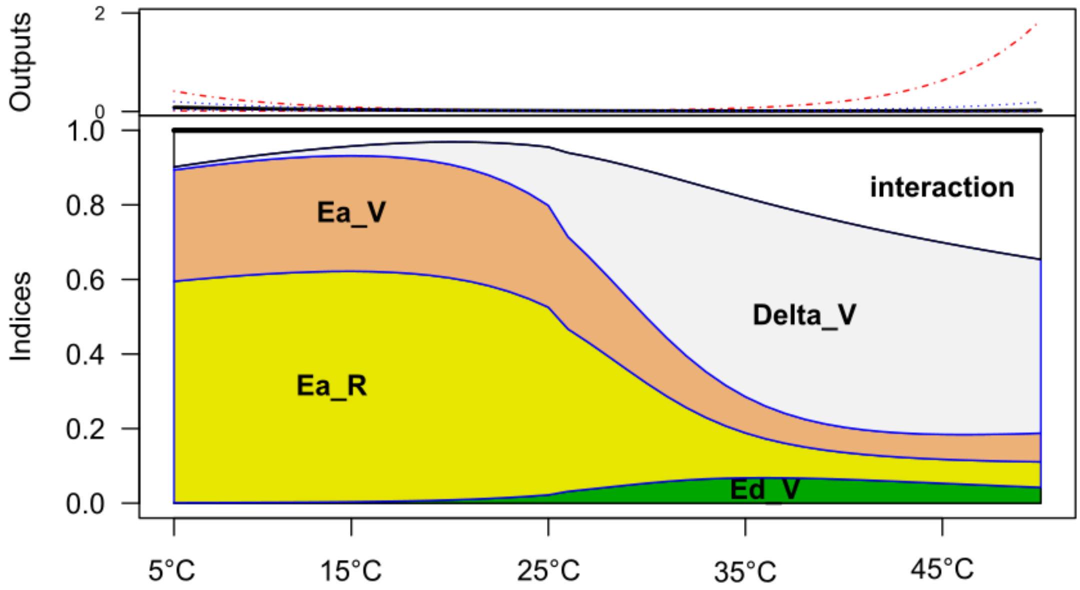

Supplementary Figure S2: Normalized partitioning of the variation of the influence of individual coefficients over model output at a broad leaf temperature range (sensitivity analysis). Extreme, inter-quartile, and median values are depicted by the dotted line, gray area, and bold line in the upper panel. Where, Ea_V, ΔS_V, and H_{dV} are respectively the activation energy, as entropy and deactivation energy of V'_{cmax}, and Ea_R is the activation energy of R_{day}

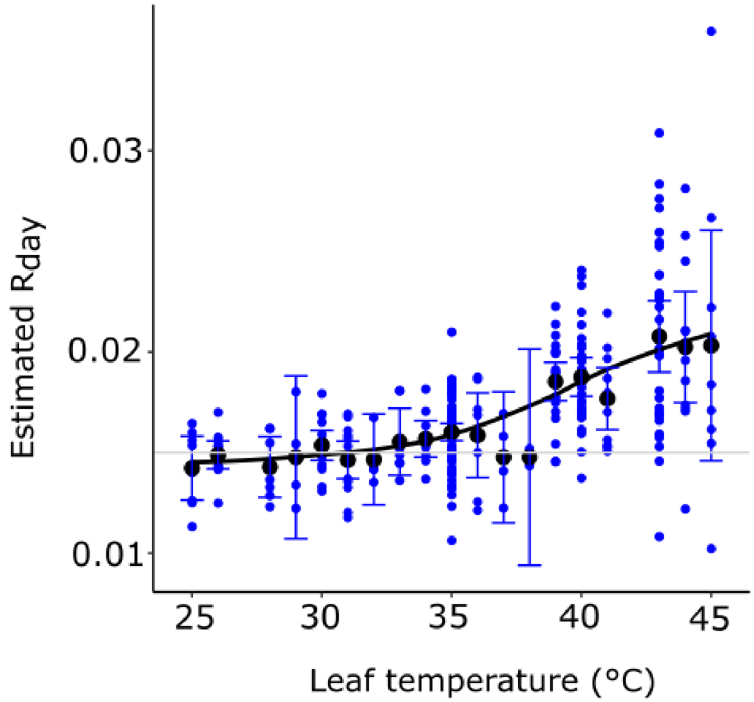

Supplementary Figure S3: Estimated R_{day} (R_{day}: V_{cmax} ratio) as a function of leaf temperature using the De Kauwe er al. (2015) model. We derived R_{day}: V_{cmax} ratio by fitting a nonlinear regression model using the 'nlsLM' function from the 'minpack.lm' package. The light-gray line is the fixed estimated R_{day}: V_{cmax} value (0.015).

References

Atkin OK, Bloomfield KJ, Reich PB, *et al.* 2015. Global variability in leaf respiration in relation to climate, plant functional types and leaf traits. New Phytologist, **206**, 614–636.

De Kauwe, MG, Lin YS, Wright IJ, *et al.* 2016. A test of the "one-point method" for estimating maximum carboxylation capacity from field-measured, light-saturated photosynthesis. New Phytologist, **210**, 1130–1144.

Kumarathunge DP, Medlyn BE, Drake JE, ***et al.*** 2019. Acclimation and adaptation components of the temperature dependence of plant photosynthesis at the global scale. New Phytologist, **22**, 768–784.

Kumarathunge DP, Medlyn BE, Duursma R. 2018. New temperature responses of V_{cmax} and J_{max}. https://remkoduursma.github.io/plantecophys/articles/new_T_responses.html. Accessed July 2022.
